# Up-regulation of immunomodulatory effects of mouse bone-marrow derived mesenchymal stem cells by tetrahydrocannabinol pre-treatment involving cannabinoid receptor CB2

**DOI:** 10.18632/oncotarget.7042

**Published:** 2016-01-27

**Authors:** Junran Xie, Dongju Xiao, Yun Xu, Jinning Zhao, Li Jiang, Xuming Hu, Yaping Zhang, Lina Yu

**Affiliations:** ^1^ Department of Anesthesiology, Sir Run Run Shaw Hospital, School of Medicine, Zhejiang University, Hangzhou, People's Republic of China; ^2^ Jiangsu Province Key Laboratory of Anesthesiology, Xuzhou Medical College, Xuzhou, People's Republic of China; ^3^ Jiangsu Province Key Laboratory of Anesthesia and Analgesia Application Technology, Xuzhou, People's Republic of China; ^4^ Department of Anesthesiology, Second Affiliated Hospital, School of Medicine, Zhejiang University, Hangzhou, People's Republic of China

**Keywords:** mesenchymal stem cells, tetrahydrocannabinol, cannabinoid receptor, inflammation, pain, Immunology and Microbiology Section, Immune response, Immunity

## Abstract

Chronic pain is commonly and closely correlated with inflammation. Both cannabinoid signaling and mesenchymal stem cells (MSCs) have been demonstrated to reduce inflammatory pain. Although cannabinoid signaling is essential for mesenchymal stem cell survival and differentiation, little is known about its role in modulatory effect of MSCs on inflammation and pain sensitivity. Here we showed that mouse bone-marrow derived MSCs (BM-MSCs) expressed both cannabinoid receptor type 1 and 2 (CB1 and CB2). CB2 expression level in BM-MSCs increased with their maturation. In addition, we found that tetrahydrocannabinol (THC) activated CB2 receptor and ERK signaling, consequently enhancing the modulation of MSCs on inflammation-associated cytokine release from lipopolysaccharides-stimulated microglia. Consistent with *in vitro* data, THC pretreatment enhanced the immunomodulatory effects of BM-MSC on thermal hyperalgesia and mechanical allodynia in chronic constriction injury model, by decreasing the release of pro-inflammation cytokines. Our study revealed the crucial role of THC in promoting the immunomodulatory effects of MSCs and proposed a new strategy to alleviate pain based on stem cells therapy.

## INTRODUCTION

*Cannabis sativa L.* is an annual, dioecious herb, belonging to the family of Cannabaceae and originating from Eastern and Central Asia [1, 2]. It has long been used as a sedative, analgesic and anti-inflammatory agent [3, 4]. Phytocann-abinoids or cannabinoids are considered the major compound responsible for the biological activities of *Cannabis sativa L*. More than 100 cannabinoids have been identified so far, among which tetrahydrocannabinol (THC), as the main psychoactive constituent of Cannabis, is the most widely studied compound [5-7].

The main receptor targets for cannabinoid signaling are type-1 and type-2 G protein-coupled cannabinoid receptors (CB1 and CB2) [8]. CB1 is widely expressed mainly at the terminal ends of central and peripheral neurons in the nervous system [9]. Once activated, CB1 is involved in the inhibition of excitatory and inhibitory neurotransmission, and can modulate cognitive, memory and motor functions, as well as analgesia [10]. CB2 is mainly expressed in the cells of the immune system, where it is commonly associated with the regulation of different immune functions including chronic inflammation of the nervous system [11]. CB2 receptors are shown to be upregulated in the central nervous system and dorsal root ganglia by pathological pain states [8, 9]. Numerous behavioral, neurochemical and electrophysiological studies have indicated CB2 as a therapeutic target for treating pathological pain states with limited centrally, mediated side effects [6, 12-14].

Multipotent mesenchymal stem cells (MSCs) have been shown to exhibit immunosuppressing properties [15]. They generate a local immunosuppressive microenvironment by secreting cytokines [16]. Some studies have documented that MSCs can release growth/neurotrophic factors as well as anti-inflammatory proteins, which modulate microglial responses to pro-inflammatory stimuli [17-19]. Moreover, single intra-brain or intravenous injections were shown to ameliorate neuro-inflammation and associated behavior in animal models of neuropathic pain, such as sciatic nerve constriction, contusion injury or spared nerve injury [20].

In this study, we investigated the role of cannabinoid THC in enhancing the immunomodulatory function of bone marrow derived mesenchymal stem cells (BM-MSCs). We studied the process in which THC promoted the immunomodulatory effect of BM-MSCs on primary microglial cultures activated by lipopolysaccharide (LPS), a standard stimulus to trigger pro-inflammatory microglial reactions. Furthermore, we used chronic constriction injury (CCI) mouse model to explore the stimulation of THC on anti-inflammatory effect of BM-MSCs.

## RESULTS

### Phenotypic characterizarion of mouse BM-MSCs

**Figure 1:**
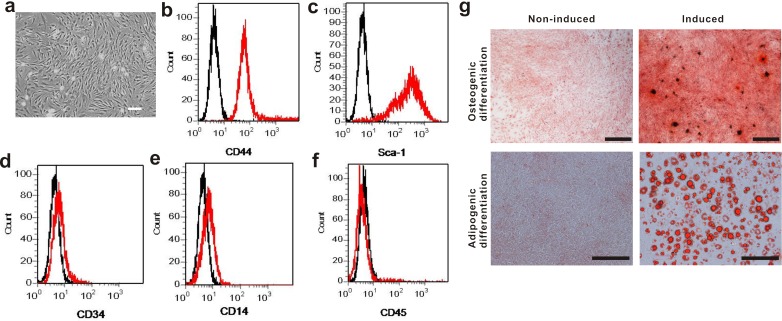
Phenotypic characterization of mouse BM-MSCs **a.** Cultured BM-MSCs after initial seeding for 5 days. Scale bar, 100 μm. **b.**-**f.** Flow cytometry analysis on BM-MSCs shows the majority of cells are CD44^+^, Sca-1^+^, CD34^−^, CD14^−^ and CD45^−^, which are characteristic phenotypes of mouse BM-MSCs. **g.** BM-MSCs was capable of differentiating into osteogenic and adipogenic lineages. Cells were stained by Alizarin red staining for osteogenic differentiation and Oil red staining for adipogenic differentiation. Scale bar, 500 μm.

The mouse BM-MSCs in culture exhibited a typical spindle-shaped morphology (Figure 1a). When they were assessed by flow cytometry, they expressed typical surface markers Sca-1 and CD44, but not CD14, CD33 or CD45, which is the characteristic phenotype of BM-MSCs (Figure 1b-1f). The differentiated cells were positive for alizarin red and oil red staining, indicating the BM-MSCs were able to differentiate into osteogenic and adipogenic lineages (Figure 1g). Taken together, the above results suggested the cultured cells are *bona fide* BM-MSCs.

### Expression of CB1 and CB2 receptors in BM-MSCs

**Figure 2:**
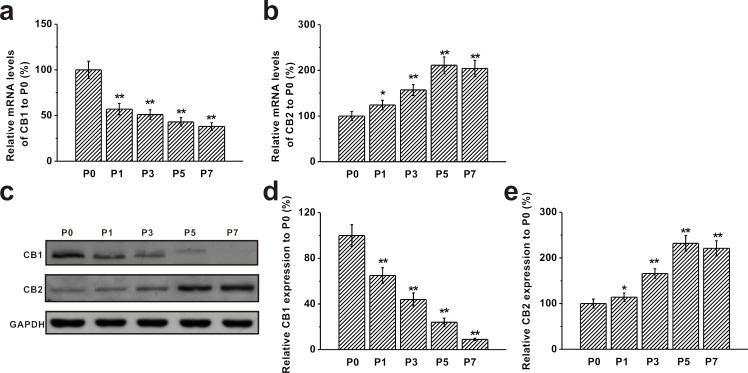
Expression of CB1 and CB2 receptors in mouse BM-MSCs **a.**-**b.** RT-PCR analysis of the mRNA levels of CB1 and CB2 receptors in MSCs from passage 0 (P0) to passage 7 (P7). Gene *GAPDH* was used as control. **c.** Western blot analysis of CB1 and CB2 protein expression in MSCs from P0 to P7. GAPDH was used as control. **d.**-**e.** Relative CB1 and CB2 protein expression in the cells normalized to those of P0. Data were presented as mean ± SEM. *p < 0.05 and **p < 0.01 *versus* P0 group.

We measured expressions of CB1 and CB2 receptors in BM-MSCs at P0, P1, P3, P5 and P7 by RT-PCR. We found that mature mRNA for both CB1 and CB2 receptors were prominently expressed on BM-MSCs. Moreover, mRNA level of the CB1 receptor was significantly decreased from P1 and continued to decline with the increase of passage numbers (Figure 2a). An opposite trend was observed for the CB2 receptor mRNA, which was markedly up-regulated with the increase of passage number and reached its maximum level at P5 (Figure 2b). Western blot analysis revealed the same trend in protein levels of CB1 and CB2 receptors at different passages. Consistent with its mRNA expression, protein level of CB1 was at the highest at P0 and gradually decreased with the increase of passage number, whereas CB2 protein level was elevated from P1 and reached the peak level at P5 (Figure 2c-2e).

### Dose-dependent effects of THC treatment on viability, proliferation and immunomodulatory effects of BM-MSCs

**Figure 3:**
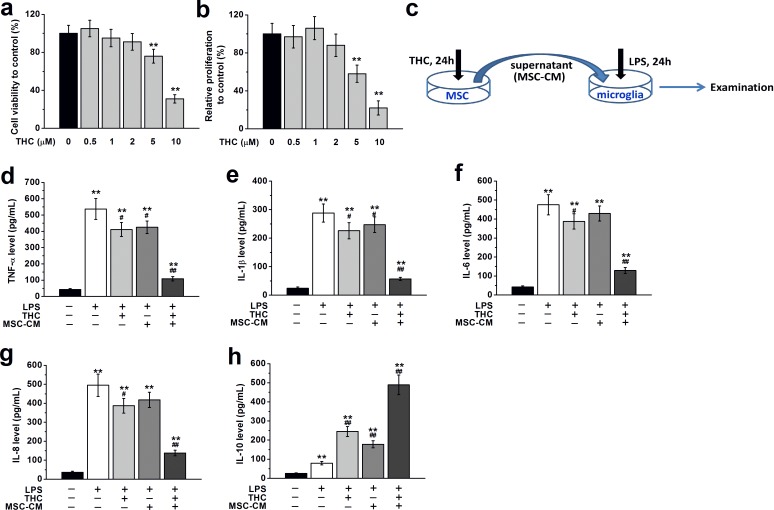
THC treatment affects viability, proliferation and immunomodulatory effects of BM-MSCs **a.**, **b.** THC treatment for 24 h at low concentration (0.5, 1 or 2 μM) had no effect on the viability and proliferation of BM-MSCs, while THC at higher concentrations (5 or 10 μM) significantly decreased cell viability and proliferation. Cell viability was measured by MTT assay. Proliferation was examined by CCK-8 assay. **p < 0.01 *versus* control (no THC treatment). **c.** Experimental diagram for MSC-CM collection and the following culture of primary microglia. Pre-treatment with 1 μM THC for 24 h significantly improved the immunomodulatory properties of BM-MSCs in primary microglia, as suggested by the lowest release of inflammatory cytokines in microglia when stimulated by LPS (100 ng/Ml, 24 h), including TNF-α **d.** IL-1β **e.** IL-6 **f.**, IL-8 **g.** and IL-10 **h.** Data were presented as mean ± SEM. **p < 0.01 *versus* control group (the first column in d-h), #*p* < 0.05 and ##*p* < 0.01 *versus* LPS group (the second column in d-h).

To explore the effects of THC on BM-MSCs, we first treated BM-MSCs with different concentrations of THC (0.5–10 μM). We found THC treatment for 24 h at low concentrations (0.5, 1 or 2 μM) had no observable effect on the viability and proliferation of BM-MSCs, while THC at higher concentrations (5 or 10 μM) significantly decreased cell viability and proliferation (Figure 3a and 3b). Therefore we used 1 μM of THC to treat BM-MSC in the rest of our study. To further identify the impact of THC on the immunomodulatory effect of BM-MSCs, we collected the supernatant of BM-MSCs treated with THC as conditional medium (CM) to culture primary microglia, and examined the expressions of inflammatory cytokines in the presence or absence of LPS (Figure 3c). Compared to control cells not grown in CM or stimulated with LPS, LPS stimulation alone significantly increased the amount of inflammatory cytokines secreted by microglia, such as TNF-α, IL-1β, IL-6 and IL-8. Microglia cultured in the presence of THC or BM-MSCs supernatant without THC treatment secreted markedly reduced levels of inflammatory cytokines when stimulated with LPS. However, the lowest release of inflammatory cytokines from microglia when stimulated by LPS was observed when they were grown in THC pre-treated BM-MSCs supernatant (Figure 3d-3g). Meanwhile, the level of anti-inflammatory cytokine, IL-10, was significantly increased by THC or BM-MSC supernatant without THC treatment, while the highest release was observed in the THC pre-treated BM-MSCs supernatant group (Figure 3h).

### Activation of CB2 receptors by THC stimulated IL-10 release and the ERK pathway in BM-MSCs

**Figure 4:**
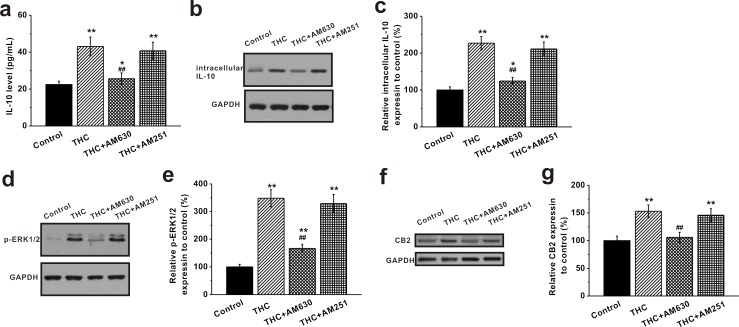
Activation of CB2 receptors by THC stimulates IL-10 release and the ERK pathway in BM-MSCs THC treatment (1 μM, 24 h) induced significant elevation of the anti-inflammatory IL-10 release **a.**, intracellular IL-10 protein expression **b.**, **c.**, and stimulated p-ERK1/2 expression **d.**, **e.** The release of IL-10 from BM-MSCs was measured by ELISA. The protein expressions of intracellular IL-10 and p-ERK1/2 were analyzed by western blot analysis and normalized to GAPDH. Meanwhile, THC treatment significantly caused CB2 receptor expression in BM-MSCs **f.**, **g.** CB2 antagonist, AM 630, greatly blocked the THC-induced elevation of IL-10 release, intracellular IL-10 expression, p-ERK1/2 expression and CB2 expression, while AM251 (CB1 antagonist) failed to. Data were presented as mean ± SEM. *p < 0.05 and **p < 0.01 *versus* control group, ##*p* < 0.01 *versus* THC group.

We further explored the potential mechanism of THC up-regulating the immunomodulatory effects of MSCs. We examined the expression of anti-inflammatory cytokine IL-10 in BM-MSCs before and after THC treatment. ELISA assay revealed that THC treatment at 1 μM for 24 h increased the release of the anti-inflammatory cytokine IL-10 from MSCs (Figure 4a). Consistently, Western blot analysis showed an increase in the intracellular IL-10 level upon THC treatment (Figure 4b, 4c). The CB2 antagonist AM630 negated the increase of both secreted and intracellular IL-10, whereas CB1 antagonist AM251 had no such effect (Figure 4a-4c). Accordingly, we found that the phosphorylated ERK1/2, known to be involved in CB2 stimulation of IL-10 release [21], was also increased in the THC-treated group, which was attenuated by AM630 but not AM251 (Figure 4d, 4e). Meanwhile, we observed an increase in the CB2 receptor expression by THC, which was reduced by its antagonist AM630 (Figure 4f, 4g). Also, the phosphorylation of Akt was greatly enhanced in the presence of THC, and PI3K inhibition by wortmannin significantly decreased Akt phosphorylation (Supplementary Figure S1).

### THC pretreatment enhanced the effects of BM-MSC on thermal hyperalgesia and mechanical allodynia

**Figure 5:**
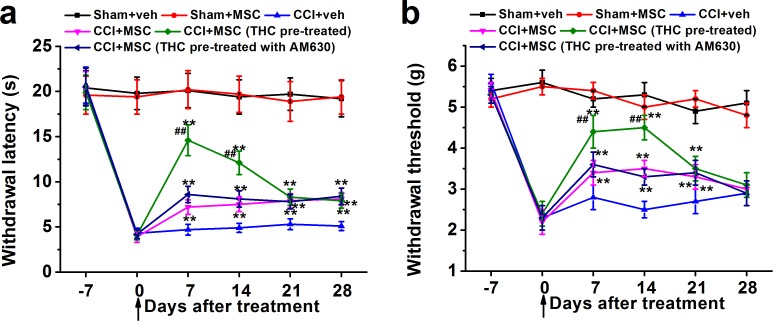
THC-pre-treated BM-MSCs significantly enhances the effects of BM-MSCs on the thermal hyperalgesia **a.** and mechanical allodynia **b.** in neuropathic mice (CCI model). BM-MSCs were pre-treated with 1 μM THC for 24 h, then administrated by intravenously injection at day 0 (indicated by the arrow). Thermal hyperalgesia was measured by Plantar test and mechanical allodynia was measured by Dynamic Plantar Aesthesiometer. Data were presented as mean ± SEM. **p < 0.01 *versus* CCI+veh group, ##*p* < 0.01 *versus* CCI+MSC (THC pre-treated) group.

In order to assess the function of THC *in vivo*, we investigated its role as therapeutic option in neuropathic pain. MSCs were administrated into the animals after pre-treated with 1 μM THC for 24 h in the presence or absence of CB2 antagonist AM630 after 7 days from sciatic injury, when neuropathic pain was already established. Pain behavior was evaluated at 0, 7, 14, 21, and 28 days after injection. Administration of THC pre-treated MSCs in the CCI mice tail vein induced a significant reduction in hyperalgesia (Figure 5a) and allodynia (Figure 5b) 7 days after injection. The effect on thermal hyperalgesia was gradually reduced from day 7, which was similar to MSC or MSC with AM630 treated CCI mice after 21 days, but still significantly different from vehicle-treated CCI at day 28 (Figure 5a). On the other hand, the effect on mechanical allodynia was maintained until day 14 and reduced to the same level as MSC or MSC with AM630 treated groups at day 21. At day 28, there was no obvious difference between all CCI mouse groups (Figure 5b). Moreover, animals were tested for anxiety-like behaviors on the elevated plus-maze. There was no significant differences in the percentage open arm entries of the total arm entries, or percentage open arm time of the total arm time between control and THC-pretreated MSCs groups (both p > 0.05, Supplementary Figure S2).

### Effects of THC pretreated BM-MSC on ipsilateral sciatic nerve cytokines

**Figure 6:**
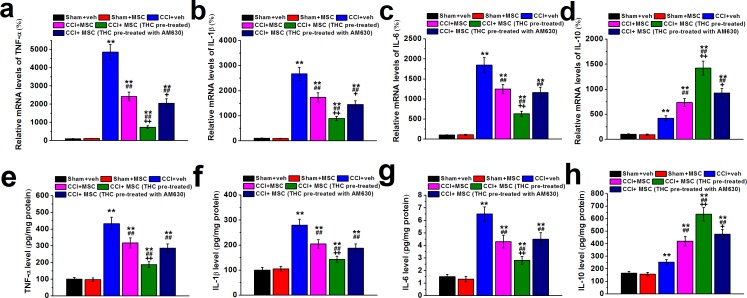
TNF-α (a and e), IL-1β (b and f), IL-6 (c and g) and IL-10 (d and h) mRNA expression and protein content in ipsilateral sciatic nerve of experimental mice 7 days after MSC administration The cytokine mRNA levels were determined by RT-PCR with GAPDH as a control and were normalized to those of sham+veh group. Cytokine protein contents were measured by ELISA and normalized to sample total protein. Data were presented as mean ± SEM. **p < 0.01 *versus* sham+veh group, ##*p* < 0.01 *versus* CCI+veh group, +*p* < 0.05 and ++*p* < 0.01 *versus* CCI+MSC group.

We next examined the mRNA and protein levels of pro-inflammatory cytokines TNF-α, IL-1β, IL-6 and anti-inflammatory cytokine IL-10. As expected, expressions of all cytokines increased significantly in CCI mice compared to control (Figure 6). However, 7 days after MSC administration, mRNA of TNF-α, IL-1β and IL-6 decreased dramatically in ipsilateral sciatic nerve of CCI mice. More importantly, THC pretreated MSC injection further decreased the mRNA levels of pro-inflammatory cytokines, which could be attenuated by CB2 antagonist AM630 (Figure 6a-6c). Interestingly, we observed an increase in IL-10 mRNA level after MSC injection, which could be enhanced by THC pretreatment whereas attenuated by AM630 (Figure 6d). Changes in the protein profiles were consistent with that of mRNA (Figure 6e-6h).

### Effect of THC pre-treatment on the percentage of BM-MSCs in the sciatic nerve

**Figure 7:**
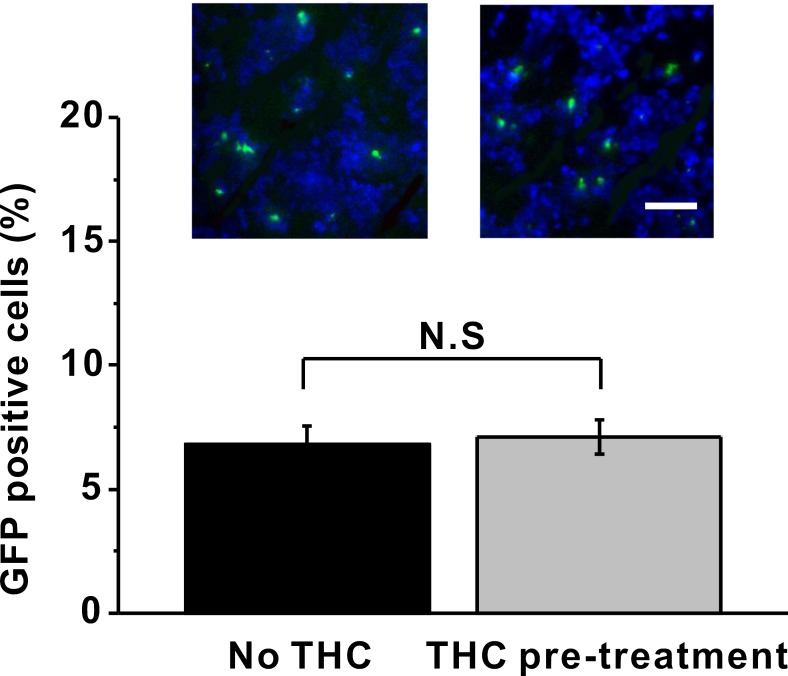
THC pre-treatment has no impact on the percentage of GFP-labeled BM-MSCs in the sciatic nerve 24 h after administration Green indicated the GFP-labeled BM-MSCs, while blue indicated the nucleus. Data were presented as mean ± SEM. N.S indicates no significance.

To exclude the possibility that THC pre-treatment may alter the percentage of BM-MSCs in the sciatic nerve after MSC injection, we isolated and sectioned the sciatic nerve and counted the GFP-labeled MSC cells with or without THC pre-treatment. We found there was no difference in percentage of GFP positive cells between these two groups (Figure 7), indicating the increase in immunomodulatory ability of BM-MSC by THC pre-treatment was not due to an increase of total number of BM-MSCs in the sciatic nerve.

## DISCUSSION

Chronic pain is common and debilitating with few effective therapeutic options [7]. It is well-known that pain and inflammation are closely correlated [13, 22]. Pain induced by tissue damage provokes the production or secretion of inflammatory mediators such as cytokines, prostanoids and neuropeptides [23]. Inflammatory mediators also produce neural effects involving activation of nociceptors, free nerve endings which act as receptors for pain [24]. There is growing literature regarding cannabinoids as potential analgesics because they have been shown to reduce inflammatory pain [5, 25-28]. Moreover, several clinical trials on THC have demonstrated significant pain reductions in patients with multiple sclerosis or chronic pain [3, 11, 29-31]. On the other hand, stem cell-based therapies also hold promise for pain treatment [32]. Transplantation of MSCs has been demonstrated to be a potentially therapeutic approach for the alleviation of chronic pain from various studies [33-35]. The MSCs relieve pain hypersensitivity through suppression of pain-related signaling cascades and reduced inflammatory cell recruitment [15]. Cannabinoid signaling is essential in regulating cell proliferation, differentiation and survival, with different outcomes depending on the molecular targets and cellular context involved [36]. Also, CB1 receptor is essential for the survival and differentiation of MSCs [9]. However, little is known about the role of cannabinoid signaling in modulatory effect of MSCs on inflammation and pain sensitivity. Here we showed that THC pre-treatment significantly enhanced the immunomodulatory effect of BM-MSCs both *in vitro* and *in vivo*.

Both CB1 and CB2 are reported to be expressed in BM-MSCs, where they play important roles in regulating their proliferation and differentiation [37, 38], which is consistent with our study that BM-MSCs expressed both CB1 and CB2 receptors (Figure 2). A significant change in endocannabinoid levels, concurrently with a sensible modification of CB2 or CB1 receptor expression, was observed during the different *in vitro* culture passages. We showed that expression of the CB2 receptor was barely detectable at the first passage and increased through the following ones, whereas CB1 expression showed the opposite trend (Figure 2). Interestingly, this inversely correlated regulation, at least in part, mimicked the same trend of receptor expression in hMSCs [39]. It has been suggested that MSCs are regulated in a way to respond to external cannabinoids via CB2 receptor activation, to perform specific tasks such as immunosuppression and anti-inflammatory action [39].

We next focused on how THC enhanced the modulation of MSCs on inflammation-associated cytokine release from LPS-stimulated microglia, the main resident immune cells in the central nerve system [18]. Consistent with previous findings, THC or MSCs alone significantly reduced the release of pro-inflammatory cytokines including TNF-α, IL-1β, IL-6 and IL-8, and increased the release of anti-inflammatory cytokines, IL-10 (Figure 3). Interestingly, the maximum effect was observed when microglia were grown in CM from MSCs pre-treated with 1 μM of THC, suggesting THC can strongly upregulate the immunomodulatory effects of MSCs. In a previous study, different concentrations of THC (0.5, 5 and 50 μM) have been tested. Although THC at 0.5 μM modestly decreased cytokine release, significant effect was only seen when THC was used at much higher concentrations [40]. Our data showed that MSCs pre-treated with THC at the concentration as low as 1 μM significantly suppressed the LPS-induced inflammation response in microglial cells. It is worth noting that THC also exhibits psychoactive effect, therefore dosage is important when considering its clinical application as anti-inflammatory reagent [41]. Strong psychotropic effects both in humans and experimental animals have been widely reported [42-46]. Adverse behavioral symptoms of THC exposure include anxiety and hypolocomotor effects [46]. In our study, THC-pretreated BM-MSCs were centrifuged and the supernatants were completely discarded to ensure no THC was administrated into the body. Also, THC pre-treated MSCs were found to have no obvious impact on anxiety-like behaviors (Supplementary Figure S2). This strategy therefore avoided the psychoactive effects of THC when used for pain relief.

We further explored the potential mechanism of THC up-regulating the immunomodulatory effects of MSCs. We found THC treatment increased both secreted and intracellular levels of IL-10 in MSCs (Figure 4a-4c). Since IL-10 has been shown to be increased by CB2 activation through the ERK 1/2 pathway [39], we also investigated the expressions of p-ERK1/2 and CB2. We found that both p-ERK1/2 and CB2 levels were increased upon THC treatment (Figure 4d, 4e). Interestingly, the increase in IL-10, p-ERK1/2 and CB2 was negated by CB2 antagonist AM630, but not CB1 antagonist AM251 (Figure 4), indicating that CB2, instead of CB1, was crucial for THC promoted anti-inflammation pathway activation in MSCs. Similar results have been reported previously in hMSCs [39]. It has been shown that stimulation of the CB2 receptor partially reversed the LPS-induced modulation of pro- and anti-inflammatory cytokines in hMSCs and directly activated the ERK2 pathway [39]. Rather than activating CB2 by agonist in LPS stimulated MSCs, our results showed that treating cells with THC induced the same effects in MSCs at physiological condition. Worthy of mentioning, many studies revealed that the activation of the PI3K/Akt pathway is linked to the neuroprotective effects of cannabinoids [47-49]. To date it is not clear whether PI3K/Akt pathway is activated by THC treatment, and our current study demonstrated that THC presence could activate PI3K/Akt pathway in MSCs, suggesting PI3K/Akt may be a common pathway for cannabinoids to exert their biological effects.

In this study, we also showed that the intravenous MSC administration ameliorated neuropathic pain symptoms using the CCI model (Figure 5), consistent with published results [50]. More importantly, THC pre-treated MSC injection induced much stronger inhibition of hyperalgesia and allodynia at early time point (7 days after injury), but the inhibitory effect was gradually reduced with the increase in time. We speculate that the up-regulation of MSC immunomodulatory effect by THC is time limited *in vivo*, and repeated administration of THC pre-treated MSCs may sustain the anti-inflammatory effect of MSCs. Further studies are needed to explore this possibility. Consistent with what we observed *in vitro*, expressions of TNF-α, IL-1β and IL-6 decreased dramatically in ipsilateral sciatic nerve of CCI mice after MSC administration, and THC pre-treatment induced further reduction. On the contrary, THC enhanced the up-regulation of IL-10 by MSCs (Figure 6). In addition, we also excluded the possibility that the increase in immunomodulatory ability of BM-MSC by THC pre-treatment was merely due to the increase of total number of BM-MSCs in sciatic nerve (Figure 7).

In conclusion, we investigated the role of THC in promoting the immunomodulatory effect of MSCs on inflammation and neuropathic pain sensitivity. We found that BM-MSCs expressed both CB1 and CB2 receptors, and THC at low concentration significantly enhanced the modulation of MSCs on inflammation-associated cytokine release from LPS-stimulated microglia. We also found the immunomodulatory effect of MSC by THC was up-regulated in CCI mouse model, where both thermal hyperalgesia and mechanical allodynia were alleviated. Taken together, our study indicated THC pre-treated MSCs as a potential therapeutic strategy in inflammation and pain treatment with more profound effects.

## MATERIALS AND METHODS

### Drugs and treatment

THC, LPS was purchased from Sigma Aldrich (St. Louis, MO, USA). THC was diluted to the final concentrations (0.5, 1, 2, 5 and 10 μM) as suggested by the instruction. LPS was dissolved in PBS containing DMSO (0.01%) to a final concentration of 100 ng/mL. The MSCs was pre-treated by THC for 24 h, while the microglia was stimulated by LPS for 24 h. AM630 (10 μM) and AM251 (10 μM) (Tocris, Avonmouth, UK) were dissolved in PBS containing DMSO (final concentration: 0.01%). Cells were treated with AM630 or AM251 for 24 hours before examination.

### CCI model

Painful neuropathy was induced on 20- to 25-g C57BL/6J male mice. CCI model for mice was described before [50]. Briefly, animals were anaesthetized with sodium pentobarbital (60 mg/kg, intraperitoneal, 0.1 ml/10g) and, under a dissecting microscope, the right common sciatic nerve was exposed at the level of the mid-thigh and, proximal to the trifurcation of the nerve; three ligatures (4/0 chromic silk, Ethicon) were loosely tied around it, at about 0.5mm spacing, until they elicited a brief twitch in the respective hind, taking care to preserve epineural circulation. Sham-operated animals (sciatic exposure without ligation) were used as controls.

### Thermal hyperalgesia and mechanical allodynia evaluation

48 mice were used in this study. Each group has 8 animals. Measurements were performed on both the ipsilateral and contralateral hind paws of all mice by researchers blind to treatments. Thermal hyperalgesia was measured as previously described using a Plantar Test Apparatus [47, 48]. Mechanical allodynia was assessed using the Dynamic Plantar Aesthesiometer, as previously described in detail [47, 48].

### BM-MSCs culture

BM-MSCs were obtained from the femurs of adult C57BL/6J mice. Bone marrow cells were incubated in α-MEM supplied with 20% FBS (Gibco, Carlsbad, CA) and 1% antibiotics (Penicillin G 10,000 units/ml, streptomycin 100 μg/ml) in the humidified CO_2_ incubator at 37oC for 5 d. The debris and suspended cells were removed and attached cells were maintained in DMEM-F12 supplemented with 10% heat inactivated FBS, 1% Glutamine, and 1% Penicillin/Streptomycin. MSCs were characterized by flow cytometry. They were also tested for their osteogenic and adipogenic differentiation ability, in order to confirm the actual mesenchymal stem cell identity. Transduction of NSCs with a lentiviral vector carrying the Green Fluorescent Protein (GFP) gene was carried as previously described [50]. After five passages of purification *in vitro*, MSCs were used for this study.

### MSC administration into CCI mice

Cell administration was always performed starting 7 days after CCI, when the pain hypersensitivity was maximal. Cultured mouse MSCs or MSCs pre-treated with THC alone or with AM630 were collected and centrifuged to get rid of THC. The cell pellets were re-suspended with medium and mechanically dissociated to a single cell suspension in phosphate-buffered saline solution with 2.5% heparin and injected intravenously into the caudal vein of mice. The amount of cells and their concentration were 1×10^6^ cells/200 μl. Both sham and CCI mice were injected with the same amount of vehicle or MSCs at the same time points. Thermal hyperalgesia and mechanical allodynia were evaluated in all mouse groups immediately before surgery and at day 7 after surgery, prior to cell injection (time 0). The subsequent evaluations were performed at days 3, 7, 14, 21, and 28 after the first MSC administration.

### Flow cytometry

MSCs were characterized by flow cytometry using PE conjugated monoclonal antibodies specific for the following antigens: CD44, Sca-1, CD34, CD14 and CD45. All antibodies were purchased from BD Biosciences (Franklin Lakes, NJ, USA). Appropriate, isotype-matched, non-reactive fluorochrome-conjugated antibodies were employed as controls. Analysis of cell populations was performed by means of direct immunofluorescence with a FACSCanto flow-cytometer (BD Pharmingen) and data were elaborated using the FACSDiva software (TreeStar Inc., Ashland, OR).

### Western blot

Total protein was extracted using RIPA lysis buffer (Cell Signaling, Irvine, CA, USA). 20 to 50 μg of protein were separated on gradient polyacrylamide-SDS gels (Applygen, Shanghai, China) and transferred to Nitrocellulose Membranes (Biorad). After blocking with TBST with 5% nonfat dry milk (Applygen) for 1 h, the membrane was incubated with antibodies against CB1 (1:200), CB2 (1:200), IL-10 (1:400) (Abcam, Cambridge, UK), p-ERK1/2 (1:200, Cell Signaling) and GAPDH (1:100, Santa Cruz, Dallas, TX, USA) overnight at 4 degree. This was followed by incubation with HRP-conjugated secondary antibodies (1:500, Pierce, Shanghai, China) for 1 h at room temperature. Antibody binding was visualized by an enhanced chemiluminescence kit according to the manufacturer's protocols (Pierce).

### Primary culture of microglia

Primary cultures were obtained from one day old C57BL/6J mice. Cerebra were dissected and meninges removed in HBSS/Hepes buffer. Brains were chopped with a razor blade. The brain pieces were put into 15 ml tube and were then centrifuged for 5 min at 3000 rpm at RT. For dissociation the pellet was incubated with trypsin (0.1%) at 37°C for 20–30 min, under shaking. DNase (0.001%) was added and the tubes were turned upside down several times. The tubes were centrifuged at 3500 rpm for 5 min at RT. The supernatant was discarded and the red blood cells were removed with a Pasteur pipette. The remaining pellet was triturated through a flame-narrowed glass pipette until a single cell suspension was obtained. The cells were seeded in DMEM (4.5 g/L Glucose, [+] L-Glutamine, [−] Pyruvate), 10%FBS, 1% Penicillin/Streptomycin onto poly-L-lysine (diluted 1:1000, Sigma, Steinheim, Germany) coated flasks. The next day, the culture medium was removed, cells were washed with PBS and new culture medium was added. Medium was changed every second day. On day 10–13, tissue culture flasks were closed tightly with parafilm and shaken for 30 min at 180 rpm at 37°C on an orbital shaker-incubator (Edmund Bühler, Hechingen, Germany) and plated on culture dishes (Nunc, Roskilde, Denmark). Microglial purity was more than 95% as determined by CD11b (AbD Serotec, Kidlington, UK) immunoreactivity.

### Conditioned medium (CM) collection and the following culture of primary microglia

MSCs were seeded at a density of 3.0×10^3^ per cm^2^. After rinsing with PBS, Neurobasal medium supplemented with 1% B27, 1% Penicillin/Streptomycin, 0.5 mM L-glutamine, and 2% FBS was added. After 24 h treatment with THC, supernatants from MSC cultures were collected, and centrifuged at 3,000 rpm for 5 min to remove remaining cells. Debris was removed by rinsing the supernatant through a 0.22 μm filter and designated as conditioned medium (CM) for primary microglia. Microglia cultured in CM was then stimulated with 100 ng/mL lipopolysaccharide (LPS, Sigma) for 24h for subsequent examination.

### MTT cell viability assay

Cell viability of BM-MSCs after THS treatment was assessed via the MTT (3-(4, 5-dimethylthiazol-2-yl)-2, 5-diphenylte-trazolium bromide) assay (Sigma), which measures the ability of cells to reduce MTT to formazan. After acid isopropanol extraction, formazan absorbance was quantified at 570 nm with a reference wavelength of 630 nm (Tecan spectrophotometer, Salzburg, Austria).

### CCK-8 cell proliferation assay

Cell proliferation rate was assayed using CCK-8 (Dojindo). 1×10^4^ BM-MSCs were seeded on 96-well culture plates (Corning, NY, USA) and treated with THS at indicated concentrations for 24 h. OD value at 450 nm was evaluated using an ELISA Reader (Promega, Madison, WI, USA) following the manufacturer's instructions.

### Osteogenic differentiation and Alizarin red staining (ARS)

BM-MSCs were plated in 24-well plates in triplicate at a density of 10×10^4^ cells/cm^2^ the previous day and then treated with osteogenic supplements (OS) consisting of DMEM supplemented with 2% FBS, 5 mM β-glycerophosphate, and 50 μM L-ascorbic acid-2-phosphate, or cultured in DMEM with 2% FBS, as a control. ARS staining was performed to evaluate the calcium deposition in cells of the osteogenic lineage obtained from BM-MSCs. Briefly, cells cultured in a 24-well plate for 21-28 days were rinsed twice with PBS, fixed with 10% v/v formalin and then stained with 1% w/v ARS solution. Orange red staining indicated the location and intensity of the calcium deposition. The presence of calcium was observed using light microscope Olympus IX71 (Olympus Corporation, Tokyo, Japan).

### Adipogenic differentiation and oil red staining

For adipogenic induction, cells were seeded in 24-well plates at a density of 30,000 cells/cm^2^. After the cells reach confluence, they were treated with adipogenic induction medium (DMEM containing 10% FBS, 1 μM dexamethasone, 60 μM indomethacin, 10 μg/ml insulin, and 0.5 mM 3-isobutyl-1-methylxanthine) for 3 days and then switched to adipogenic maintenance medium (growth medium plus 10 μg/ml insulin) with media replaced every other day. The formation of adipocytes was evaluated by oil red staining. Briefly, cells were washed with PBS and incubated with 10% formalin for 1h. Cells were then washed with distilled water and 60% isopropanol and dried completely, followed by Oil Red O working solution (Sigma) incubation for 10min. The solution was removed and cells were washed with water before monitored by microscope.

### RNA extraction and real-time RT-PCR

The RNA isolation, reverse transcription, and PCR analysis were performed. In brief, total cellular RNA was isolated using TRIzol reagent (Invitrogen Corp., Pleasanton, CA, USA), 1 μg of RNA was reverse transcribed using TaqMan Reverse Transcription Reagents (Applied Biosystems, Waltham, MA, USA), and the mRNA levels of the indicated genes were analyzed in triplicate using SYBR Green master mixture (Applied Biosystems) and a Chromo-4 real time RT-PCR instrument (MJ Research, St. Bruno, Quebec, Canada). The mRNA levels were normalized to GAPDH (internal control) and gene expression was presented as -fold changes (ΔΔCt method).

### Enzyme-linked immunosorbent assay (ELISA)

The protein expression of TNF-α, IL-1β, IL-6, IL-8 and IL-10 was determined by the corresponding ELISA kit (R&D Systems, Minneapolis, MN, USA) according to the instructions. To measure the cytokine release in cells, the supernatants were collected and centrifuged, and then the cell-free supernatants were used for ELISA assay. To measure the cytokine release in ipsilateral sciatic nerves, the nerve samples were homogenized in 0.40 ml of ice-cold phosphate-buffered saline containing a protease inhibitor cocktail (Sigma) and centrifuged at 10,000 g for 15 min, and the supernatant was used for ELISA assay. Cytokine concentrations were determined by interpolation with standard curves assayed on individual plates and normalized to protein content in each sample.

### Statistical analysis

Data are reported as the mean ± SEM of the number of experiments indicated in each case. One-way ANOVA followed by a Student Newman-Keuls *post hoc* test was used to determine the statistical significance between groups. For comparisons between relevant treatments, an unpaired Student's *t-*test was performed.

## SUPPLEMENTARY MATERIAL FIGURES


