# Supplementary material for: Up-regulation of immunomodulatory effects of mouse bone-marrow derived mesenchymal stem cells by tetrahydrocannabinol pre-treatment involving cannabinoid receptor CB2
Source: Oncotarget. 2016 Jan 27;7(6):6436–47. doi: 10.18632/oncotarget.7042 (PMC4872725; doi:10.18632/oncotarget.7042)
Supplement: Supplementary file 1 [file oncotarget-07-6436-s001.pdf]

Up-regulation of immunomodulatory effects of mouse bone-marrow derived mesenchymal stem cells by tetrahydrocannabinol pre-treatment involving cannabinoid receptor CB2

Supplementary Material

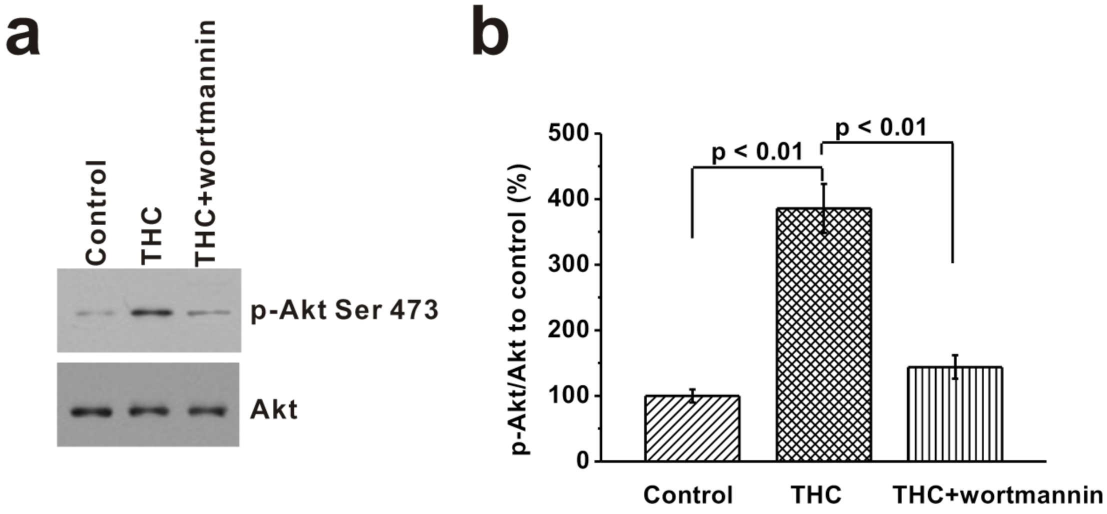

Figure S1. Effect of THC on the phosphorylation of Akt, detected by western blotting (a). Wortmannin was used to inhibit PI3K pathway. Relative p-Akt to total Akt was shown in (b). Data were presented as mean±S.D.

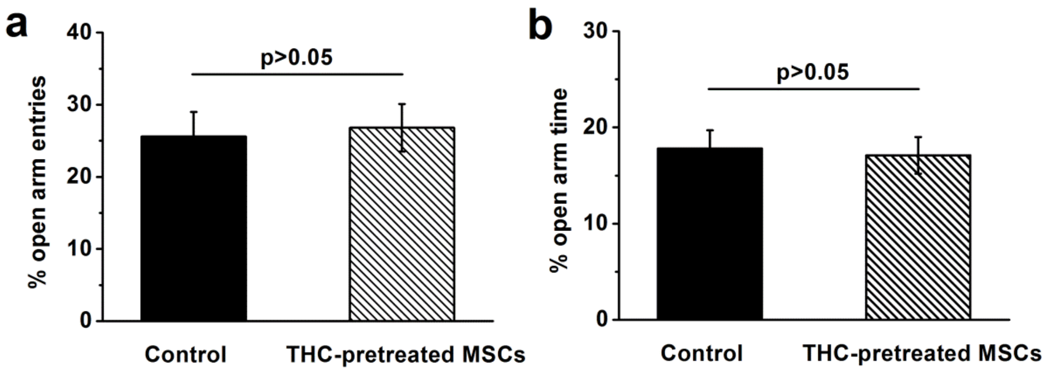

Figure S2. Anxiety-like behaviors of animals administrated by THC-pretreated MSCs, measured by the elevated plus-maze (EPM). Two parameters were selected and observed: % open arm entries (a) and % open arm time (b). Data were presented as mean±S.D. n = 8 for each group.
